# Effect of High-intensity Training and Probiotics on Gut Microbiota Diversity in Competitive Swimmers: Randomized Controlled Trial

**DOI:** 10.1186/s40798-022-00453-8

**Published:** 2022-05-10

**Authors:** Viktor Bielik, Ivan Hric, Simona Ugrayová, Libuša Kubáňová, Matúš Putala, Ľuboš Grznár, Adela Penesová, Andrea Havranová, Sára Šardzíková, Marián Grendar, Eva Baranovičová, Katarína Šoltys, Martin Kolisek

**Affiliations:** 1 Department of Biological and Medical Sciences, Faculty of Physical Education and Sport, Comenius University in Bratislava, 814 69 Bratislava, Slovakia; 2 Department of Outdoor Sports and Swimming, Faculty of Physical Education and Sport, Comenius University in Bratislava, 814 69 Bratislava, Slovakia; 3 Biomedical Center, Institute of Clinical and Translational Research, Slovak Academy of Sciences, 845 05 Bratislava, Slovakia; 4 Department of Microbiology and Virology, Faculty of Natural Sciences, Comenius University in Bratislava, 842 15 Bratislava, Slovakia; 5 Biomedical Center Martin, Jessenius Faculty of Medicine in Martin, Comenius University in Bratislava, 036 01 Martin, Slovakia; 6 Comenius University Science Park, Comenius University in Bratislava, 841 04 Bratislava, Slovakia

**Keywords:** Gut microbiome, Physical exercise, Athletes, Butyrate, Probiotics

## Abstract

**Background:**

Physical exercise has favorable effects on the structure of gut microbiota and metabolite production in sedentary subjects. However, little is known whether adjustments in an athletic program impact overall changes of gut microbiome in high-level athletes. We therefore characterized fecal microbiota and serum metabolites in response to a 7-week, high-intensity training program and consumption of probiotic Bryndza cheese.

**Methods:**

Fecal and blood samples and training logs were collected from young competitive male (*n* = 17) and female (*n* = 7) swimmers. Fecal microbiota were categorized using specific primers targeting the V1–V3 region of 16S rDNA, and serum metabolites were characterized by NMR-spectroscopic analysis and by multivariate statistical analysis, Spearman rank correlations, and Random Forest models.

**Results:**

We found higher α-diversity, represented by the Shannon index value (HITB-pre 5.9 [± 0.4]; HITB-post 6.4 [± 0.4], *p* = 0.007), (HIT-pre 5.5 [± 0.6]; HIT-post 5.9 [± 0.6], *p* = 0.015), after the end of the training program in both groups independently of Bryndza cheese consumption. However, *Lactococcus spp*. increased in both groups, with a higher effect in the Bryndza cheese consumers (HITB-pre 0.0021 [± 0.0055]; HITB-post 0.0268 [± 0.0542], *p* = 0.008), (HIT-pre 0.0014 [± 0.0036]; HIT-post 0.0068 [± 0.0095], *p* = 0.046). Concomitant with the increase of high-intensity exercise and the resulting increase of anaerobic metabolism proportion, pyruvate (*p*[HITB] = 0.003; *p*[HIT] = 0.000) and lactate (*p*[HITB] = 0.000; *p*[HIT] = 0.030) increased, whereas acetate (*p*[HITB] = 0.000; *p*[HIT] = 0.002) and butyrate (*p*[HITB] = 0.091; *p*[HIT] = 0.019) significantly decreased.

**Conclusions:**

Together, these data demonstrate a significant effect of high-intensity training (HIT) on both gut microbiota composition and serum energy metabolites. Thus, the combination of intensive athletic training with the use of natural probiotics is beneficial because of the increase in the relative abundance of lactic acid bacteria.

**Supplementary Information:**

The online version contains supplementary material available at 10.1186/s40798-022-00453-8.

## Key Points

- The current study is a randomized controlled trial evaluating the effect of high-intensity training (HIT) on the bacterial composition in the gut of high-level swimmers.
- The study indicates that high-intensity swimming during the pre-competition period can increase bacterial diversity.
- The positive effect of HIT on gut microbiota can be increased by regular consumption of natural probiotics.

## Introduction

A healthy lifestyle and diet are key components of a healthy gut [1]. In addition, physical exercise itself brings benefits such as the enrichment of microflora diversity, the growth of beneficial bacteria, and the development of a commensal population [2, 3]. Differences in the intestinal microbiome between athletes, obese subjects, and controls have been previously documented [4, 5]. Athletic training with a characteristic structure in terms of volume and intensity of exercise leads to "healthier" intestinal microbiota [6]. High-intensity interval training (HIIT) brings beneficial changes in the gut microbiota in mice [7, 8], previously inactive human adults [9], and healthy college students [10]. A significant association between changes in training structure over six weeks and bacterial composition has been reported in collegiate swimmers [11]. Interestingly, shorter lasting HIIT is reported to have no effect on the overall bacterial diversity or community structure of lean and overweight men [12] and women [13]. Changes in the abundance of several bacterial taxa can even be observed after a one-day extreme endurance event (163 km mountain footrace) [14]. However, the largest changes caused by a one-day event are related to metabolites (short-chain fatty acids—SCFA) produced by intestinal bacteria rather than to massive changes in microbiota structure. [15]. In this regard, probiotics are a known and relatively well-studied intervention involved in the production of SCFA [16]. Moreover, the positive effects of dairy and non-dairy fermented foods and of lactic acid bacteria (LAB) on human health through the modulation of the gut microbiome are well documented [17–19]. We have recently reported the effect of traditional Slovakian fermented sheep cheese, among many probiotic foods, on the composition of gut microbiota [13].

The training pattern of competitive swimmers prior to the season’s best performances is characterized by progressive increases in the volume and frequency of high exercise intensity [20]. However, little is known as to whether an “athlete’s gut”, which has been accustomed to years of physical exercise, might further benefit from adjustments in the athletic program prior to a competition event. Therefore, the purpose of this study was to compare the gut microbiome and metabolome of young swimmers before and after the completion of seven weeks of their pre-competition training program. Based on our recent research with naturally fermented foods, one of our goals was to determine whether the consumption of probiotic Bryndza cheeses can bring additional benefits to the gut microbiome and metabolic variables [13].

## Methods

### Recruitment and Study Population

The data were obtained in a longitudinal prospective study registered on ClinicalTrials.gov under No: NCT02325804, conducted in Biomedical Research Center, in Bratislava, Slovakia. The project was approved by the Ethics Committee of Bratislava Self-Governing Region No.05239/2016/HF. This study was executed in conformity with the principles from the Declaration of Helsinki for experiments involving human beings. After reading the written informed consent and after an explanation of the particular steps of the study and discussion with investigators, signed informed consent was obtained from all subjects before their participation in the study. The study was also approved by the Ethics Committee of the Faculty of Physical Education and Sports at Comenius University (FTVS UK-3/21).

### Subjects

We recruited, during the 2019 season, male (*n* = 17) and female (*n* = 7) swimmers competitive at the national level and between the ages of 16–25 years of age. The athletes came from two swimming clubs. We randomly divided athletes into two groups, i.e., those with “only” high-intensity training (HIT) and those with high-intensity training and the use of probiotic cheese (HITB). Exclusive criteria set to avoid skewed microbial data were as previously described [13].

### Study Protocol

Study subjects were asked to undergo 12 h of fasting and to avoid intensive exercise 24 h before examination. Examination started in the morning at 08:00 a.m. in the outpatient clinic of internal medicine and diabetes at the Institute of Clinical and Translational Research, Biomedical Research Center, Slovak Academy of Sciences, Bratislava. First, we took the medical and personal history of the subjects, and thereafter, we measured their body weight, height, body fat percentage, amount of fat mass, and fat-free mass by using bioelectrical impedance (Omron 511BF, OMRON HEALTHCARE Co., Ltd. Kyoto, Japan). Waist and hip circumference were also measured. The body mass index (BMI) was calculated. Blood pressure was measured via the arm during at least 5 min of rest (OMRON). Blood was drawn in the fasting state into polyethylene tubes containing ethylenediaminetetraacetic acid (EDTA) as the anticoagulant and immediately cooled in ice or into polyethylene tubes without anticoagulant (to obtain serum). After centrifugation at 4 °C, all plasma and serum aliquots were stored at − 70 °C until assayed.

Fecal samples were collected by participants in a DNA/RNA Shield-Fecal Collection Tube to maintain the stability of the nucleic acids in the stool specimens (ZymoResearch, Irvine, CA, USA). Participants had previously been instructed in the collection of specimens at home. Blood and fecal samples were collected twice: once before and once after the training period. DNA was extracted from each stool, stored on ice, and immediately frozen at − 80 °C.

### Acquisition of Clinical, Exercise, and Dietary Data

#### Physical Exercise

All enrolled athletes from both the HIT and HITB groups completed a 7-week training program at the time of the season when the athletes were expected to be peaking for performance. The last week ended with the Slovak Swimming National Championship over a long course (pool 50 m in length).

#### Intervention: Diet with Probiotics

We instructed all athletes to follow their normal diet. We additionally administered probiotic sheep cheese "Bryndza" to 12 athletes (HITB) at a frequency of 3–4 times a week and at a dose of 30 g for the entire 7 weeks of the training program. Samples of Bryndza were stored for microbial analysis when a new batch of cheese was received. Bryndza is rich in natural probiotics and contains 13 families, 24 genera, and 44 species [21]. We report the structure of Bryndza microbiome in Additional file 1.

### Microbial Analysis

Total DNA from the stool samples was extracted using the ZymoBiomics DNA/RNA mini kit (ZymoResearch Scientific, Irvine, CA, USA) in accordance with the manufacturer’s protocol. DNA was amplified by specific primers targeting the V1-V3 regions of 16SrDNA. Amplicons were used for the preparation of DNA libraries and sequenced using the Illumina MiSeq platform by 300 bp pair-end reads (Illumina, San Diego, CA, USA). Details of this DNA sequencing procedure can be found in Hric et al [13].

### Illumina Data Processing

Illumina Data Processing Adapters and low-quality read ends were removed using Geneious (Biomatters, Ltd., Auckland, New Zealand) based on quality control statistics generated by FastQC [22]. The paired reads were merged (set as paired reads), and the 3´ ends of reads were trimmed (error probability limit: 0.03). Microbial profiles of samples were assessed by comparison with RDP and the Silva database.

### Blood Plasma Metabolites; NMR Data Acquisition

Selected plasma metabolite concentrations were obtained using nuclear magnetic resonance (NMR) analysis as described in Hric et al*.* [13]*.*

The concentrations of the following 20 plasma metabolites were analyzed by NMR: lactate, alanine, valine, leucine, isoleucine, glucose, acetate, 3-OH-butyrate, acetone, pyruvate, phenylalanine, tyrosine, glutamine, lysine, histidine, tryptophane, keto-leucine, keto-isoleucine, and ketovaline, and lipoprotein fractions containing fractions of VLDL, LDL, IDL, and HDL.

### 25-OH Vitamin D Concentrations

Serum 25-OH vitamin D concentrations were determined with a chemiluminescent microparticle immunoassay (CMIA; ARCHITECT 25-OH vitamin D: Abbott Laboratories Diagnostics Division Abbott Park, IL 60064 USA) diagnostic system in a certified hospital laboratory (SYNLAB Bratislava, Slovakia).

### Statistical Analysis

The data were explored and analyzed by R ver. 4.0.3 [23], by the use of the rstatix [24], ggpubr [25], and randomForestSRC [26]. Exploratory data analysis involved the visualization of data by swarmplots overlaid with boxplots. Differences of values before and after treatment were normalized by the Inter-Quartile Range (IQR) to facilitate visual explorations of the data within the groups. Gross outliers were excluded from the data (usually 0 or 1 observation per variable). Normality of the resulting data was assessed by the Quantile plot with the 95% confidence band, constructed by bootstrap. The null hypothesis of the zero mean difference was tested by the Welch t test. The *p* values were corrected for multiple hypothesis testing by the Benjamini Hochberg correction. An analogous procedure was utilized for comparisons between groups, if the differences were not IQR-normalized. Random Forest (RF) machine learning algorithm was trained to predict before/after status in the HIT and HITB group. In each RF, the feature selection was performed by nested cross-validation, with the graph depth as the objective function. Performance of RF with the selected features was assessed by the out-of-bag ROC curve and quantified by the Area under ROC (AUC). Correlations between the characteristics of the gut microbiota and metabolism were analyzed using the Spearman correlation coefficient. The significance level of all statistical analyses was set at 0.05. Power calculations for this study were based on our previous study of the effect of probiotic intake on *Lactococcus* abundance [13] by using an on-line calculator [27]. The study required a sample size of 5 to achieve a power of 80% and a level of significance of 5% (two-sided) for detecting a mean of the differences 0.0567 between pre- and post-intervention, assuming the standard deviation of the differences to be 0.0224. ClustVis was used to visualize multidimensional data by using principal component analysis (PCA) [28].

## Results

### Training Variables

This training program undertaken during intervention was built on a previous training cycle and was characterized by a progressively increased volume and frequency of high exercise intensity. High-intensity training consisted of swimming lengths of 12.5, 25, 50, and 100 m at an intensity of > 90% of maximum speed. For swimming lengths of 12.5 m, we used 2–4 series, repeated 12 times, with a swimming parachute regularly on Mondays. For 25 m lengths, we used 2 series with 16 repetitions on Wednesday. For 50 m lengths, we used 4–8 repetitions and for 100 m 2–8 repetitions. These latter training sessions most often fell on a Friday. The volume of training gradually decreased, and the number of repetitions in the main set increased from Phase1 to Phase 2A. Phase 2A, high-intensity swimming, was followed by Phase 2B characterized by a taper during which training volume was exponentially reduced towards the Slovak Swimming National Championship over a long course. The detailed yardage and intensity distribution of the training undertaken by the athletes is presented in Table 1.

**Table 1:** Training variables before (Phase 1) and during (Phase 2A and 2B) intervention. Total swimming distance (TS), high-intensity swimming distance (HIS), and training hours out of the water (OUT) are presented as mean and standard deviation

	TS km/wk	HIS km/wk	HIS to TS (%)	OUT h/wk
Phase 1	45.9 ± 14.4	4.8 ± 2.8	10.4 ± 10.0	2.5 ± 0.7
Phase 2A	31.3 ± 5.5	7.2 ± 3.9	20.7 ± 6.9	2.6 ± 0.9
Phase 2B	22.1 ± 3.9	3.2 ± 1.3	14.3 ± 13.2	1.8 ± 0.4

Phase 1 (from March 18 to May 3, 2019), experimental Phase 2A (from May 6 to June 7, 2019), and experimental Phase 2B (from June 8 to June 15, 2019) which towards to Slovak Swimming National Championship in Long course (15–16th June 2019, Žilina, Slovakia). All measurements were taken before Phase 2A and repeated after Phase 2B (in the week following the championship)

### Microbial Analysis of Stool

Eleven bacterial phyla were detected in the two groups: (a) high-intensity training (HIT) and (b) high-intensity training and use of probiotic cheese (HITB). The highest percentages of abundance in the HIT group were seen for phyla *Firmicutes* (80.2%—HIT-pre; 76.3%—HIT-post), *Bacteroidota* (17.7%—HIT-pre; 21.6%—HIT-post), and *Actinobacteriota* (0.99%—HIT-pre; 1%—HIT-post). The phyla *Proteobacteria*, *Verrucomicrobiota*, *Cyanobacteria*, *Desulfobacterota*, *Fusobacteriota*, *Fibrobacterota*, *Patescibacteria*, and *Campylobacterota* were detected with an abundance of less than 1%. In the group HITB, we observed predominantly the phyla *Firmicutes* (82.3%—HITB-pre; 77.7%—HITB-post), *Bacteroidota* (14.1%—HITB-pre; 19.9%—HITB-post), and *Actinobacteriota* (2.1%—HITB-pre; 1.1%—HITB-post). *Proteobacteria*, *Verrucomicrobiota*, *Cyanobacteria*, *Campylobacterota*, *Desulfobacterota*, and *Patescibacteria* were present with an abundance of less than 1% in the HITB group.

We detected 62 families in two groups. In the samples from subgroup HITB-pre, we detected 51 families, and in the subgroup HITB-post, 47 families. The subgroup HIT-pre included 41 families, and the HIT-post, 58 families. The dominant family in both groups was the *Lachnospiraceae* (HITB-pre—47.6%; HITB-post—45.4%; HIT-pre—41.5%; HIT-post—43.5%) followed by the families *Ruminococcaceae*, *Bacteroidaceae*, *Prevotellaceae*, and *Oscillospiraceae*.

In all, 185 genera were identified, of which 147 genera were in group HITB (147- HITB- pre; 137—HITB-post) and 160 genera in group HIT (136—HIT-pre; 160—HIT-post). The 10 most abundant genera identified in each group were *Faecalibacterium*, *Blautia*, *Bacteroides*, *Roseburia*, *Subdoligranulum*, *Ruminococcus*, *Prevotella*_9, *Agathobacter*, *Coprococcus*, and the [*Ruminococcus*] torques group. The significant within-group differences in the relative abundance of bacterial populations are shown in Table 2 (HITB) and Table 3 (HIT).

**Table 2:** Bacterial taxa (phyla, order, family, genus) before and after 7 weeks of the high-intensity training phase with probiotics

Taxa (%)	HITB-pre (*n* = 12)	HITB-post (*n* = 12)	*p *value
*Bacteroidetes*	11.1495 (± 5.9885)	15.7330 (± 5.2891)	0.026
*Bacteroidia*	10.7238 (± 5.9885)	15.5264 (± 5.2891)	0.026
*Bacteroidales*	10.7238 (± 5.9862)	15.5264 (± 5.2913)	0.026
*Christensenellales*	0.5273 (± 0.4799)	0.3936 (± 0.4001)	0.041
*Lactobacillales*	0.0706 (± 0.0758)	0.1578 (± 0.1747)	0.015
*Bacteroidaceae*	7.095 (± 4.4728)	9.7773 (± 4.7393)	0.053
*Muribaculaceae*	0.0395 (± 0.0737)	0.4395 (± 1.3809)	0.051
*Rikenellaceae*	0.4123 (± 0.3741)	0.8465 (± 0.7732)	0.050
*Streptococcaceae*	0.0688 (± 0.0756)	0.1559 (± 0.1747)	0.019
*Christensenellaceae*	0.5273 (± 0.4799)	0.3936 (± 0.4001)	0.041
*Veillonellaceae*	0.0089 (± 0.0168)	0.0126 (± 0.0201)	0.080
*Bacteroides*	7.0955 (± 4.4728)	9.7773 (± 4.7393)	0.053
*Alistipes*	0.3838 (± 0.3881)	0.6861 (± 0.5919)	0.060
*Lactococcus*	0.0021 (± 0.0055)	0.0268 (± 0.0542)	0.008
*Christensenellaceae R7 group*	0.5273 (± 0.4809)	0.3936 (± 0.4006)	0.041
*Fusicatenibacter*	2.1098 (± 1.6001)	1.2069 (± 7212)	0.028

Data are presented as means and standard deviations. *HITB-pre* variables before high-intensity training with probiotics, *HITB-post* variables after high-intensity training with probiotics

**Table 3:** Bacterial taxa (phyla, order, family, genus) before and after 7 weeks of the high-intensity training phase without probiotics

Taxa (%)	HIT-pre (*n* = 12)	HIT-post (*n* = 12)	*p* value
*Bacteroidetes*	13.7922 (± 6.6851)	17.6415 (± 8.8349)	0.050
*Negativicutes*	0.0671 (± 0.1210)	0.1141 (± 0.1840)	0.019
*Gammaproteobacteria*	0.2082 (± 0.1836)	0.4174 (± 0.4210)	0.023
*Burkholderiales*	0.2057 (± 0.1859)	0.3987 (± 0.4088)	0.023
*Barnesiellaceae*	0.1109 (± 0.0859)	0.1848 (± 0.1456)	0.041
*Rikenellaceae*	0.4061 (± 0.2757)	0.8471 (± 0.7084)	0.010
*Veillonellaceae*	0.0376 (± 0.0609)	0.0632 (± 0.1039)	0.050
*Sutterellaceae*	0.2057 (± 0.1859)	0.3978 (± 0.4039)	0.023
*Adlercreutzia*	0.0122 (± 0.0161)	0.0403 (± 0.0492)	0.008
*Butyricimonas*	0.0065 (± 0.0094)	0.0196 (± 0.0238)	0.028
*Alistipes*	0.3961 (± 0.2772)	0.8025 (± 0.7105)	0.010
*Holdemanella*	0.0061 (± 0.0174)	0.0646 (± 0.1725)	0.028
*Lactococcus*	0.0014 (± 0.0036)	0.0068 (± 0.0095)	0.046
*Incertae Sedis*	0.0000 (± 0.0000)	0.0045 (± 0.0068)	0.043
*Eubacterium eligens group*	0.0713 (± 0.0658)	0.1776 (± 0.1693)	0.012
*Parasutterella*	0.0634 (± 0.0663)	0.1364 (± 0.1657)	0.033

Data are presented as means and standard deviations. *HIT-pre* variables before high-intensity training without probiotics, *HIT-post* variables after high-intensity training without probiotics

**Fig. 1:**
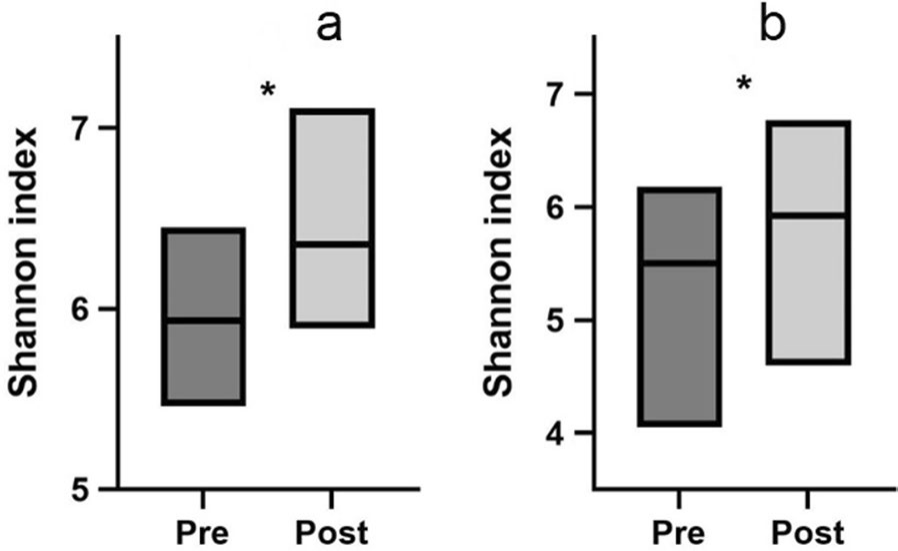
α-diversity by Shannon index before and after intervention. **a** High-intensity training and use of probiotic cheese (HITB); **b** High-intensity training only (HIT). Floating bars are the minimum to maximum values, the line shows the mean. *p < 0.05

In the α-diversity analysis, we identified a significant increase of OTUs after both intervention programs (HITB-pre 1939.0 [± 421.4]; HITB-post 2367.9 [± 421.4], *p* = 0.001), (HIT-pre 1395.4 [± 447.1]; HIT-post 2155.5 [± 603.0], *p* = 0.000). Further, we detected a significant increase of the Shannon index value after both intervention programs (HITB-pre 5.9 [± 0.4]; HITB-post 6.4 [± 0.4], *p* = 0.007), (HIT-pre 5.5 [± 0.6]; HIT-post 5.9 [± 0.6], *p* = 0.015) (Fig. 1). The value of the Simpson index did not differ between the groups.

At the phylum level, we observed a significant increase in the relative abundance of *Bacteroidiota* (*p*(HITB) = 0.0260; *p*(HIT) = 0.0050) after both interventions.

**Fig. 2:**
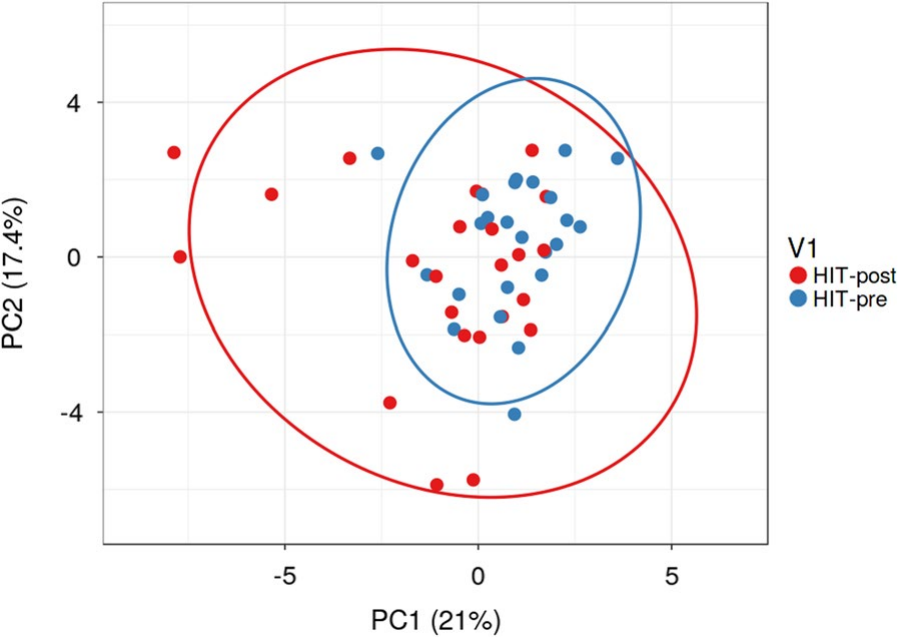
β-diversity of analyzed samples represented by significantly altered (*p* < 0.05) bacterial taxa, selected by Random Forest machine learning analysis, before and after 7 weeks of the high-intensity training phase (HIT and HITB) as visualized by PCA. SVD with imputation was used to calculate principal components. The *X* and *Y* axis show principal component 1 and principal component 2 that explain 21% and 17.4% of the total variance, respectively. Prediction ellipses are such that, with a probability of 0.95, a new observation from the same group will fall inside the ellipse (*n* = 48 data points). HIT-*pre* variables before high-intensity training, HIT-*post* variables after high-intensity training

In our microbial analysis of lactic acid bacteria (LAB), we detected a significant difference of the order *Lactobacillales* (*p* = 0.015*)* and of the family *Streptococcaceae* (*p* = 0.019) between the HITB-pre and the HITB-post groups, the latter group showing higher levels than the former. Furthermore, the genus *Lactococcus* was significantly increased after both intervention programs (*p*(HITB) = 0.008; *p*(HIT) = 0.046). However, the genus *Lactococcus* increased after the HITB program 128-fold, whereas in the HIT intervention, only a 5-fold elevation of relative abundance was seen. We also found a few bacterial shifts in the relative abundance of bacteria producing SCFA metabolites. *Butyricimonas* (*p* = 0.028) and *Alistipes* (*p* = 0.010) were increased after the HIT intervention program. Similarly, the genus *Alistipes* was increased after HITB intervention, although the difference was not significant (*p* = 0.060). Selected bacterial genera enabled the discrimination of subjects from the pre and post HITB groups by using principal component analysis (PCA) (Fig. 2).

### Biochemical Analysis

There were no significant changes in blood parameters such as total cholesterol, insulin, or glucose after the interventions. However, we detected a significant decrease of uric acid (*p* = 0.003) and alanine transaminase (ALT; *p* = 0.002) levels in the HITB-post group compared with the HITB-pre group after probiotic intervention. Moreover, an increase of the sodium (Na^+^; *p* = 0.054) level was observed in the HITB program.

After both interventions, we identified a significant increase of the vitamin D level (HITB-pre 16.14 [± 3.18]; HITB-post 36.21 [± 6.27], *p* = 0.000), (HIT-pre 19.49 [± 5.31]; HIT-post 39.15 [± 8.55], *p* = 0.000). Further, significant differences in the creatinine levels were observed in both programs: we detected an increase of the metabolite after HIT (*p* = 0.001) and a decrease after HITB intervention (*p* = 0.009).

### NMR analysis of Plasma Metabolites

We observed significant differences in the relative concentrations of certain metabolites after the interventions. In the intra-group comparison, we detected a significant increase of lactate (*p*(HITB) = 0.000; *p*(HIT) = 0.030) and pyruvate (*p*(HITB) = 0.003; *p*(HIT) = 0.000) concentrations after both intervention programs.

Moreover, after both programs, we identified significant differences in the concentrations of certain SCFA metabolites. We found a significant decrease of acetate after both programs (*p*(HITB) = 0.000; *p*(HIT) = 0.002). Furthermore, butyrate concentration was significantly decreased in the HIT program (*p* = 0.019). After HITB intervention, a decrease of butyrate concentration was detected, although the difference was not significant (*p* = 0.091).

### Random Forest Machine Learning

**Fig. 3:**
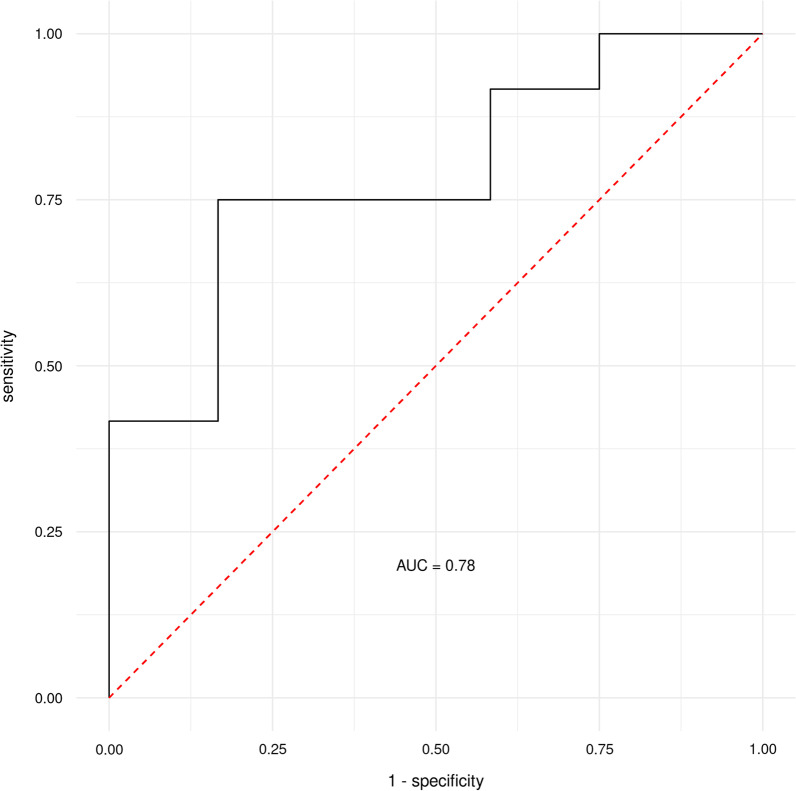
ROC (receiver operating characteristic) curves with an area under the ROC curve (AUC) for the RFM-L algorithm acetate, pyruvate, *Butyricimonas*, butyrate, *Bacteroidetes*, *Alistipes*, and α-diversity (Shannon index) as joint predictors/discriminators between pre- and post-intervention in HIT. FPR, false-positive rate; HIT, high-intensity training group; RFM-L, Random Forest machine-learning; TPR, true positive rate

**Fig. 4:**
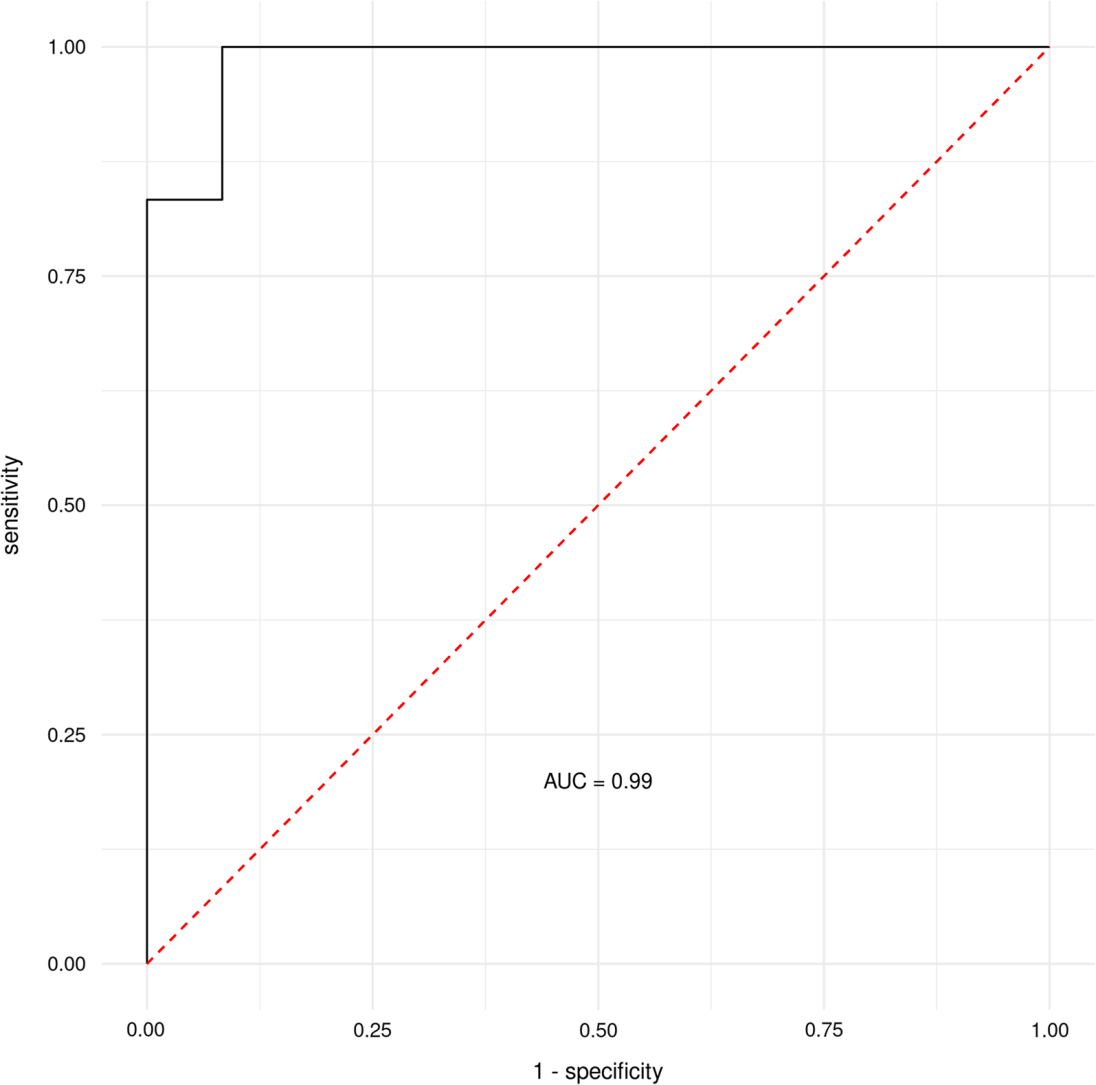
ROC (receiver operating characteristic) curves with an area under the ROC curve (AUC) for the RFM-L algorithm pyruvate, lactate, acetate, α-diversity (Shannon index), and butyrate, as excellent joint predictors/discriminators between pre- and post-intervention in HITB. FPR, false-positive rate; HITB, high-intensity training and use of probiotic cheese group; RFM-L, Random Forest machine-learning; TPR, true positive rate

The Machine Learning (ML) analysis with acetate, pyruvate, *Butyricimonas*, butyrate, *Bacteroidetes*, *Alistipes*, and α-diversity (Shannon index) used as joint predictors of the before/after status in the HIT group led to an ROC curve with an AUC of 0.78, which represents a fair set of variables for discriminating subjects from the pre- and post-HIT group. (Fig. 3). Furthermore, the ML analysis with pyruvate, lactate, acetate, α-diversity (Shannon index), and butyrate used as joint predictors led to an ROC curve with an AUC of 0.99, which represents an excellent set of variables for discriminating subjects from the pre- and post-HITB group (Fig. 4).

## Discussion

The reported controlled trial was used to study the effect of 7 weeks of a high-intensity exercise program, with or without probiotic Bryndza cheese consumption, on the gut microbiota composition and metabolomics in high-level competitive swimmers. We hypothesized that intensive physical training would have a positive influence on SCFA producers in the gut and on metabolites in the blood of athletes. We expected that regular consumption of Bryndza cheese would have an additional positive effect on the relative abundance of lactic acid bacteria (LAB). Our main findings demonstrated that high-intensity exercise increased bacterial diversity as measured by the Shannon index in both groups. Furthermore, we found a higher abundance of the LAB after Bryndza consumption (*Lactobacillales* and *Lactococcus*). Remarkably, we observed a higher relative abundance of intestinal SCFA producers. In addition, we found a decrease in serum butyrate and acetate in both groups.

Effects of athletic training and physical fitness on increased microbial diversity has been widely published based on human and animal models [3, 14, 29–32]. However, the reported results related to α-diversity differ. In our recently published study, we reported no higher α-diversity in elderly athletes compared with controls, despite lifelong systematic endurance training and higher cardiorespiratory fitness [4]. Similarly, Allen et al. [33] did not observe statistical differences between the α-diversity of lean and of obese participants at baseline or after 6 weeks of endurance-based exercise training [33]. Moreover, Resende et al. [34] reported no differences in α-diversity in 24 previously sedentary men after 10 weeks of aerobic exercise training. Therefore, our observation of an increasing α-diversity after the training program implemented for 7 weeks prior to the season’s best performances is of interest. At first glance, our results are not in agreement with a previous study undertaken on collegiate swimmers [11]. Hampton-Marcell et al. [11] observed a significant decrease in overall microbial diversity during peak training through the season’s best performances of the swimmers. They reported a decline of training volume at the final phase (last 2 weeks) with an average yardage of 11.3 ± 8.1 km/week; the decrease in the volume of training occurred in parallel with a decrease in α-diversity [11]. However, the swimmers in our study covered, during the last two weeks before competition, on average a weekly yardage that was twice as long as that reported in the study of Hampton-Marcell et al. [11]. We assume that the length of training program and the intensity explain the controversy related to the effects of physical exercise on α-diversity. Whereas short-lasting HIT (3–4 weeks) had no effect on the overall bacterial diversity of lean and overweight men [12] and women [13], a longer intervention in animal models improved gut microbial diversity [7, 8].

The largest intestinal changes induced by physical training concern both the increase in number of SCFA producers and the production of energetic metabolites (e.g., SCFA) [6]. As found in professional international rugby union players, microbial-derived SCFAs (including acetate, propionate, and butyrate) and metabolic pathways are enhanced in the athletes relative to controls [32]. We have however observed only a few changes related to the types of butyrate producers. Here, we report the increase in the relative abundance of the genus *Butyricimonas spp.* and of *Alistipes spp.* at the end of the training period. Concomitantly, we have also noticed decreases in serum metabolites (acetate, butyrate). Ketone oxidation (e.g., β-hydroxybutyrate (βHB)) might provide an alternative, energetically advantageous fuel for skeletal muscle contraction [35]. However, βHB oxidation contributes minimally to energy expenditure, although the large relative contribution of exogenous βHB oxidation occurs during light exercise [36]. Ingestion of ketone salts (βHB) prior to cycling has been established to improve fat oxidation during steady-state exercise at 30%, 60%, and 90% ventilatory threshold but impairs high-intensity exercise performance [37]. The results from trained cyclists suggest that the pre-training ingestion of βHB has no benefit for endurance performance [38, 39]. The acute exogenous intake of ketone ester can even slightly impair short high-intensity endurance exercise performance [40]. The production of ketone bodies, while providing an alternative substrate for oxidative phosphorylation, actually decreases muscle glycolysis and plasma lactate concentrations [35]. Here, we report an increased intensity of exercise accompanied by a higher rate of anaerobic glycolysis during the peak period. This might explain the higher baseline pyruvate and lactate as a consequence of muscle adaptation to an exhaustive training period. As observed previously, baseline serum pyruvate is increased in response to a period of high-intensity interval training [41]. Additionally, we consider that the reported changes in serum metabolites, namely in acetate, butyrate, lactate, and pyruvate, are the result of skeletal muscle metabolism rather than of the metabolism of gut microbiota.

An additional purpose of the study was to find out whether high-intensity exercise training combined with Bryndza sheep cheese consumption can change the relative abundance of LAB in the collected stools of athletes. Similarly to Pangallo [42], we report that, among other LAB, the incidence was highest of the genus *Lactococcus* spp. (86% of all identified taxa). After 7 weeks of intervention with Bryndza consumption, the athletes exhibited an increased relative abundance of *Lactococcus spp*. However, we also report higher *Lactococcus spp.* in swimmers who have not consumed Bryndza. This can be explained by the seasonal variation of vitamin D that has been previously positively associated with *Lactococcus spp*. [43, 44]. In confirmation of this idea, we have found a positive association between the relative abundance of *Lactococcus spp*. and vitamin D in the blood. At the beginning of the study (end of April), both groups of swimmers were deficient in vitamin D [45], perhaps because of the lack of vitamin D synthesis from the low levels of sunlight during the winter months. Subsequently, lighter clothing and exposure of more of the body to sunlight probably increased vitamin D synthesis, as indicated by our reported elevated concentration of vitamin D in blood by the end of June. Remarkably, the *Lactococcus spp*. in the Bryndza group increased 13-fold, whereas in the group without Bryndza intervention, we observed "only" a 5-fold positive change in relative abundance.

The RF and ML analyses have identified pyruvate, acetate, butyrate, and α-diversity (Shannon index) as suitable joint predictors with a fair (HIT) and excellent (HITB) ability to discriminate between subjects pre- and post-intervention. Furthermore, the GraphDepth analyses ranked pyruvate, lactate, and acetate as the three best-discriminating factors for the subjects within the HITB group, and acetate, pyruvate, Butyricimonas and butyrate as the four best-discriminating factors for the subjects within the HIT group. Hence, they can be proposed as suitable joint biomarkers discriminating between high-volume/low-intensity and low-volume/high-intensity training phases with and/or without probiotic use.

Although controversy exists in the literature relating to the accuracy and validity of self-reported dietary intake as estimated by Food Frequency Questionnaires, 24-h dietary interviews, and dietary records [46, 47], the first limitation of our study is the lack of reports the athletes’ dietary data. Further, we did not measured stool metabolites and therefore were unable to confirm the effects of any significant increase of intestinal SCFA producers at the end of the training period. In particular, the relative rather than absolute concentrations of metabolites is a limitation of the study. Moreover, the distribution of men and female subjects and effects of the menstrual cycle might have influenced our results. Despite non-significant changes of body weight during intervention, we might have missed alterations in body fat and fat-free mass percentage. The athletes were from two different swimming clubs and were trained by two coaches. Although the coaches worked closely together and planned the training for this study, we admit that each coach has their own signature. Finally, our study participants were all swimmers, and so the results of our study cannot be translated to other high-level athletes in general.

A remarkable strength of our investigation is that the cohort of experienced high-level athletes shared the same training program, freedom from non-communicable disease, and being medication naïve. The identification of suitable joint predictors with a fair (HIT) and excellent (HITB) ability to discriminate between subjects pre- and post-intervention by Random Forest machine learning algorithm strengthens our analysis and distinguishes the intervention effect between HIT and HITB.

## Conclusion

The pre-competition training program characterized by an increased volume of high-intensity exercise improved intestinal α-diversity independently from the consumption of a natural probiotic. Serum metabolites (lactate, pyruvate, acetate, and butyrate) reflect the structure of the training period with an emphasis on anaerobic metabolism. Bryndza cheese consumption in combination with intensive athletic training brings additional probiotic benefits by increasing the amount of LAB.

## Supplementary Information

**Additional file 1:** Taxonomic profiling of bacterial communities in probiotic Bryndza cheese.

## Data Availability

The data presented in the study are deposited in the GenBank repository with BioProject accession number SAMN23561538 - SAMN23561590, Bioproject PRJNA785427.
